# Severe acute pancreatitis 5 years after pancreaticoduodenectomy: A case report

**DOI:** 10.1016/j.ijscr.2019.07.045

**Published:** 2019-07-22

**Authors:** Yuya Ashitomi, Shuichiro Sugawara, Ryosuke Takahashi, Koki Ashino, Toshihiro Watanabe, Osamu Hachiya, Wataru Kimura

**Affiliations:** a First Department of Surgery, Yamagata University Graduate School of Medical Science, Yamagata, Japan; b Department of Surgery, Touto Kasukabe Hospital, Saitama, Japan

**Keywords:** PD, pancreaticoduodenectomy, CT, computed tomography, Acute pancreatitis, Pancreaticoduodenectomy, Necrosectomy, Case report

## Abstract

**Introduction:**

Acute pancreatitis is a known complication of pancreaticoduodenectomy (PD). However, no reports in the literature describe a late delayed severe acute pancreatitis. We report a case of acute pancreatitis 5 years after PD in a patient who needed intensive care for his complication.

**Presentation of case:**

A 64-years-old man presented with upper abdominal pain and reported a history of PD 5 years prior to presentation. Contrast-enhanced computed tomography revealed an edematous pancreatic remnant with inflammation of the surrounding tissue, and he was diagnosed with acute pancreatitis. His condition worsened, and he was transferred to our hospital the following day. He was admitted to the intensive care unit to manage respiratory and circulatory insufficiency. Although his condition improved, an abdominal abscess was identified, and necrosectomy was performed on day 43 of hospitalizaiton. We carefully removed as much necrotic tissue as was possible without injury to the pancreaticojejunal anastomosis and the ascending colon. Inflammation gradually subsided, and he was discharged on day 111 of hospitalization. The last drain was removed in day 133 of admission to our hospital. Pancreatitis and abdominal abscess have not recurred until the time of writing this paper.

**Discussion:**

Delayed severe acute pancreatitis is rare. Necrosectomy can treat an abdominal abscess; however it is important to avoid injury to other organs.

**Conclusion:**

Clinicians should be aware that severe acute pancreatitis can occur after PD.

## Highlights

• Acute pancreatitis is one of complication of pancreaticoduodenectomy.
• Delayed severe acute pancreatitis which needs intensive care is very rare.
• Necrosectomy effective treated abdominal abscess, but it is important to avoid injury to surrounding organs.
• Clinicians should be aware that severe pancreatitis chould occur even after pancreatectomy.

## 1 Introduction

Acute pancreatitis is a known complication of pancreaticoduodenectomy (PD). Only a few reports have described delayed acute pancreatitis [1], although several previous studies have reported pancreatitis in the acute phase after PD [[2], [3], [4]]. Notably, no case report has described delayed severe acute pancreatitis. We present a rare case of severe acute pancreatitis, which occurred 5 years after PD. This work has been reported in line with the SCARE criteria [5].

## 2 Presentation of case

**Fig. 1:**
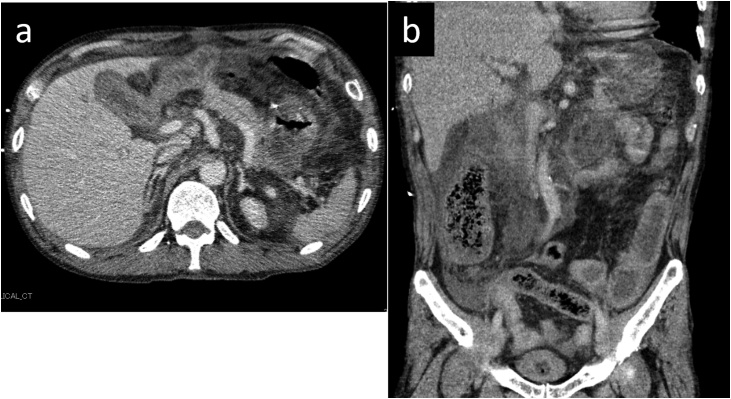
Contrast-enhanced CT scan showing spread of inflammation throughout the abdomen. (a) Axial view, (b) Coronal view.

A 64-years-old man presented with pain in his epigastric region. He reported a history of PD (modified Child’s reconstruction) for a pancreatic neuroendocrine tumor 5 years prior to presentation. Contrast-enhanced computed tomography (CT) showed an edematous pancreatic remnant with inflammation of the surrounding tissue. He was diagnosed with acute pancreatitis and received nafamostat mesilate and ulinastatin. His condition worsened despite treatment, and he was transferred to our hospital the following day. Laboratory investigations showed elevated serum pancreatic enzymes (pancreatic amylase 190 U/L, lipase 312 U/L). Abdominal CT revealed the spread of inflammation throughout the abdomen (Fig. 1). He was admitted to the intensive care unit for the management of respiratory and circulatory insufficiency. He needed intensive care with intubation, catecholamine administration, and blood purificaiton treatment. His condition gradually improved, and he was discharged from the intensive care unit on day 20 of hospitalization. However, significant inflammation persisted.

**Fig. 2:**
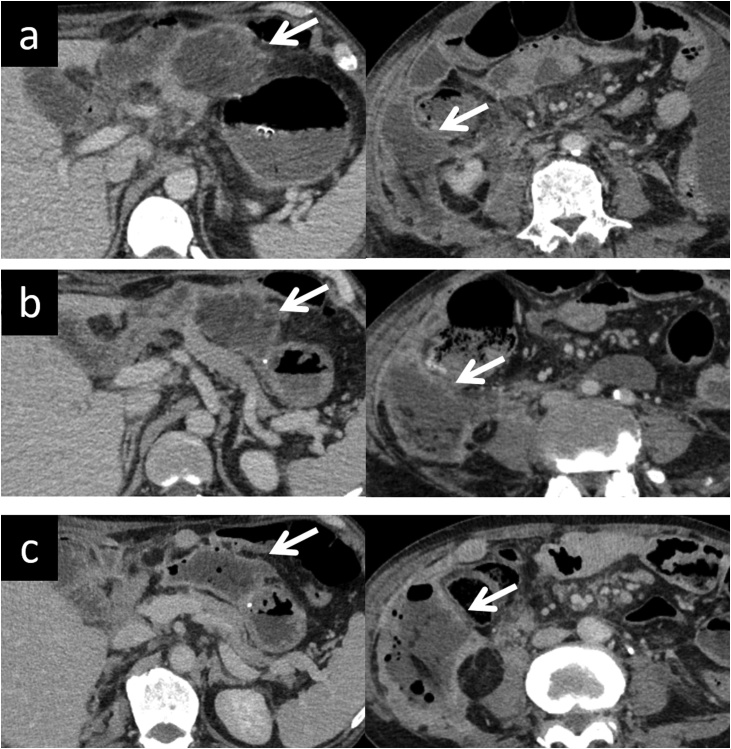
Abdominal CT scan obtained on (a)day16, (b)day23, and (c)day42 of hospitalization showing gradual transformation of ascites into an abdominal abscess (arrow).

**Fig. 3:**
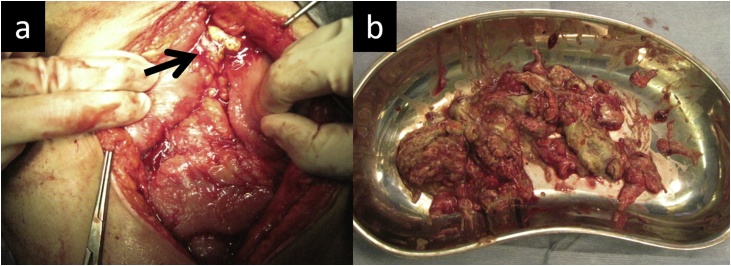
(a)Intraoperative findings showing removal of the abscess located anterior to the site of the pancreaticojejunostomy (arrow) and right paracolic gutter. (b)Image showing the removed necrotic tissue.

After pancreatitis improved, ascites was observed in the right paracolic gutter and anterior to the site of the pancreaticojejunostomy. We performed necrosectomy on day43 of hospitalization because ascitic fluid infection led to abscess formation (Fig. 2). We carefully removed as much necrotic tissue as was possible, without injury to the pancreaticojejunal anastomosis and ascending colon (Fig. 3). Although the drains were retained over a prolonged period (owing to continued drainage), inflammation gradually improved. The abscess in the right paracolic gutter recurred after drain removal. Percutaneous puncture was performed on day 104 of hospitalization. He was discharged on day 111 of hospitalization with 1 drain in place, which was removed on day 133. Pancreatitis and abdominal abscess have not recurred until the time of writing this report.

## 3 Discussion

The incidence of acute pancreatitis is 5–80 per 100,000 person-years, which varies between countries. High alcohol intake and gallstones are primary etiological contributors to acute pancreatitis [6]. A survey performed in Japan showed that the mortality rate of severe acute pancreatitis was 10.1% [7]. The mortality rate of pancreatitis associated with multi-organ failure is significantly high [8,9]. The Atlanta classification categorize acute pancreatitis into interstitial edematous and necrotizing pancreatitis [10]. This patient presented with interstitial edematous pancreatitis.

Acute pancreatitis could occur after PD, and a few reports in the literatures have described this condition. An observational study has reported a case of postoperative pancreatitis that occurred immediately after the PD and was associated with postoperative pancreatic fistula formation [2,3]. Delayed pancreatitis is commonly attributed to a pancreaticoenteric anastomotic stricture [11]. Only a few studies have reported delayed acute pancreatitis, and notably, no reports discuss delayed severe acute pancreatitis. Yen et al. reported delayed acute pancreatitis diagnosed in 23 of 539 patients undergoing PD. All patients with delayed acute pancreatitis showed mild disease, and only 15 patients presented with a pancreaticoenteric anastomotic stricture [1].

A pancreaticoenteric anastomosis stricture was not identified in our patient, and pancreatitis could be attributed to alcohol intake prior to admission. Although this patient did not show necrotizing pancreatitis, managing the abdominal abscess was challenging in this case. Necrosectomy effectively treated the abscess; However it is important to avoid injury to surrounding organs during this procedure. We ensured that we did not injure the pancreaticoenteric anastomosis in this patient.

## 4 Conclusion

Clinicians should be aware that severe pancreatitis could occur even after pancreatectomy.

## Funding

No funding.

## Ethical approval

Institutional ethics committee approval was taken for the publication (no. 2019-S-5).

## Consent

Written informed consent was obtained from the patients for publication of this case report and accompanying images. A copy of the written consent is available for review by the Editor-in-Chief of this journal on request.

## Author contribution

Yuya Ashitomi: writing paper, data collection and literature review.

Shuichiro Sugawara: the surgeon who did the operation.

Ryosuke Takahashi: helped treat this patient and reviewing this paper.

Koki Ashino: helped treat this patient and reviewing this paper.

Toshihiro Watanabe: treat this patient and reviewing this paper.

Osamu Hachiya: reviewing and final approval of this paper.

Wataru Kimura: reviewing and final approval of this paper.

## Registration of research studies

There is no need to register because it is a case report.

## Guarantor

Osamu Hachiya.

## Provenance and peer review

Not commissioned, externally peer-reviewed

## Declaration of Competing Interest

No conflict of interest.
